# Airborne Bacterial Interactions: Functions Out of Thin Air?

**DOI:** 10.3389/fmicb.2015.01476

**Published:** 2015-12-22

**Authors:** Bianca Audrain, Sylvie Létoffé, Jean-Marc Ghigo

**Affiliations:** Genetics of Biofilms Laboratory, Department of Microbiology, Institut PasteurParis, France

**Keywords:** bacterial volatile compounds, metabolism, signaling, bacterial interactions, biofilm, antibiotic resistance

## Abstract

Bacteria produce and release a large diversity of small molecules including organic and inorganic volatile compounds, hereafter referred to as bacterial volatile compounds (BVCs). Whereas BVCs were often only considered as wasted metabolic by-product sometimes perceived by animal olfactory systems, it is increasingly clear that they can also mediate cross-kingdom interactions with fungi, plants and animals. Recently, *in vitro* studies also reported the impact of BVCs on bacterial biology through modulation of antibiotic resistance, biofilm formation and virulence. Here, we review BVCs influence on bacterial adaptation to their environment and discuss the biological relevance of recently reported inter- and intra-species bacterial interactions mediated by BVCs.

## Introduction

**FIGURE 1:**
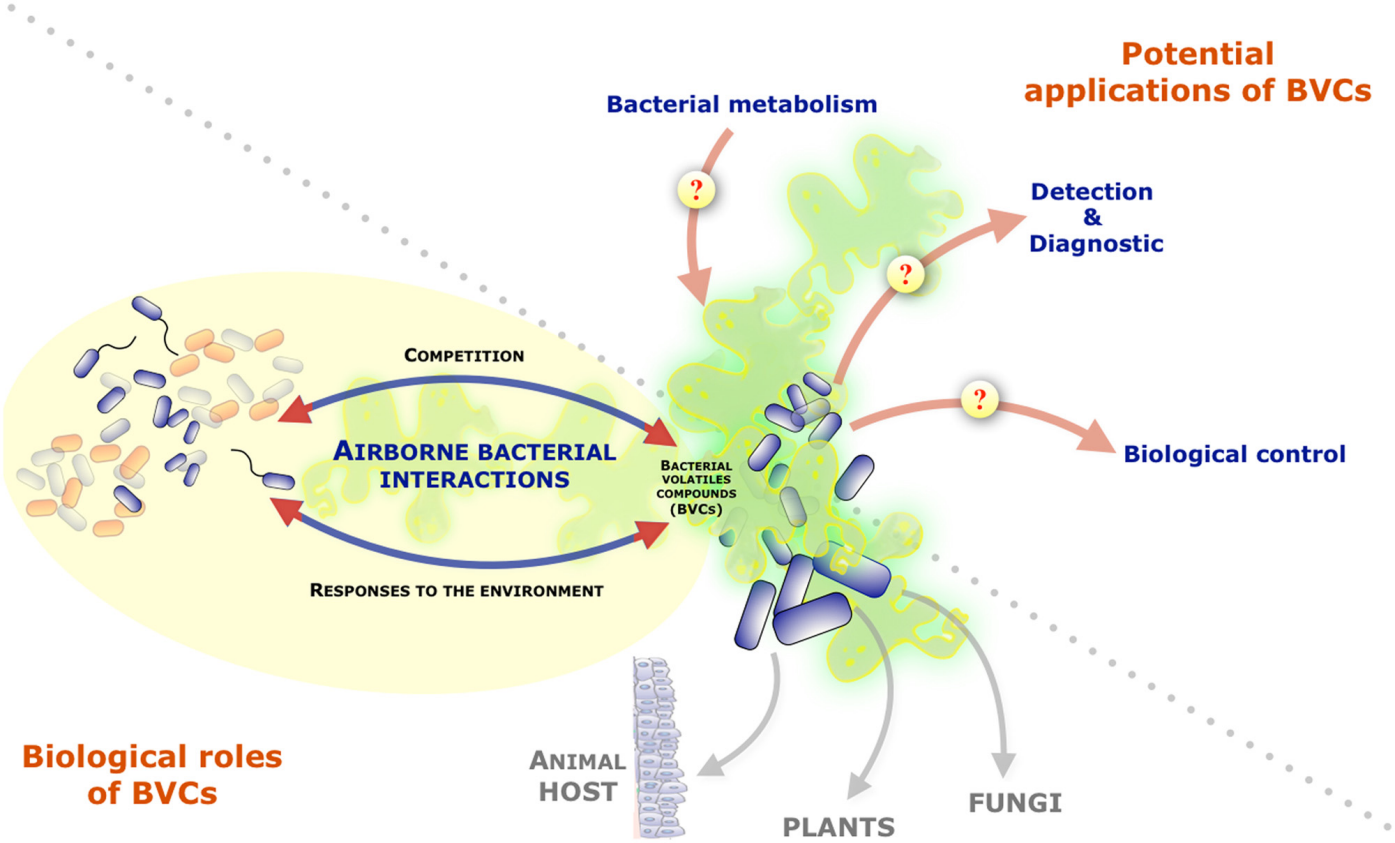
**Role of volatile compounds released by bacteria and their potential interests and applications.** Graphical representation of the focus of the review on bacteria-bacteria interactions mediated by bacterial volatile compounds (BVCs). The figure also mentions BVCs impact on other organisms and illustrates the potential interests/applications of the study of BVCs as indicators of bacterial metabolism and pool of molecules with diagnostic or biocontrol potentials.

While the contribution of diffusible soluble secondary metabolites in bacterial ability to communicate, compete or cooperate with neighboring microorganisms has been actively investigated, bacteria also produce and release a wide diversity of volatile compounds that can be readily detected in the bacterial headspace (Schulz and Dickschat, 2007). Nevertheless, the potential biological role(s) of organic and inorganic bacterial volatile compounds or gases (BVCs) was often overlooked. Recent studies, however, demonstrated that they could mediate a variety of interactions between bacteria and their environment. Indeed, several BVCs were shown to influence growth and differentiation in fungi, to induce systemic resistance against bacterial pathogens in plants or to affect behaviors in invertebrates (**Figure 1**; Gallagher and Manoil, 2001; Ryu et al., 2003; Kai et al., 2008, 2009; Niu et al., 2010; Effmert et al., 2012). In addition of their action on a wide range of eukaryotic organisms, several reports also revealed the potential impact of BVCs on bacteria themselves (Audrain et al., 2015). This review will present the current knowledge on BVCs influence on inter- and intra-species bacterial interactions and will discuss their biological relevance and the interest to further study this particular class of bacterial metabolites.

## Role of BVCs in Bacterial Competition

Bacteria often compete for space, nutrients or others resources through production of metabolic by-products providing them with an advantage over surrounding bacteria. Several BVCs display a direct negative effect, as it is the case for some volatile compounds emitted from rhizosphere bacteria *Bacillus*, *Pseudomonas*, *Serratia*, or *Streptomyces* affecting bacterial growth. For instance, dimethyl disulfide emitted from *Pseudomonas fluorescens* and *Serratia plymuthica* displays bacteriostatic effects against two plant bacterial pathogens, *Agrobacterium tumefaciens* and *A. vitis* (Dandurishvili et al., 2011). Moreover, albaflavenone produced by *Streptomyces* sp. exhibits antibacterial activity against *Bacillus subtilis* (Gurtler et al., 1994).

Some soluble short-chain fatty acids (acetate, succinate, propionate, or isobutyrate) are also able to inhibit growth of several enteropathogens (*Salmonella enteritidis*, *S. typhimurium* and *Escherichia coli*) (Hinton, 1995), and also growth and sporulation of *Clostridium perfringens* (Wrigley, 2004). Although these experiments were performed using short-chain fatty acids in solution, these metabolites are produced by *Veillonella* species or *Bacteroides fragilis* (Hinton, 1995) and several other members of the intestinal microbiota (Effmert et al., 2012) suggesting that volatile short-chain fatty acids could also play a role in control of competing commensals and also enteropathogens in the intestinal tract.

Some BVCs are also able to modulate at a distance the production of antimicrobials. Indeed, volatile compounds produced by *Collimonas pratensis* increased production of secondary metabolites in *P. fluorescens* that showed antimicrobial activity against *Bacillus* sp (Garbeva et al., 2014). In *P. aeruginosa*, the production of molecules with antimicrobial activity such as pyocyanin seems also to be influenced by volatile compounds (Venkataraman et al., 2011, 2014). A recent study reported that 2,3-butanediol, produced by co-habitant fermenter bacteria such as *S. marcescens* enhances production of *P. aeruginosa* pyocyanin exhibiting antimicrobial activity, which then could help *P. aeruginosa* to occupy a niche, especially in cystic fibrosis lungs (Venkataraman et al., 2014); 2,3-Butanediol and its volatile precursor 2,3-butanedione have thus been detected in airways of cystic fibrosis patients (Whiteson et al., 2014). All these study therefore suggest a potential direct and indirect role of BVCs in bacterial competition.

## Volatile-Dependent Bacterial Responses to the Environment

Several studies described BVCs as potential airborne chemical cues modulating gene expression, membrane permeability or enzyme activation resulting in alteration of bacterial behaviors. For instance, *P. fluorescens* transcriptional response differs upon exposure to volatiles emitted by rhizospheric bacteria such as *C. pratensis* and *S. plymuthica*, including dimethyl disulfide and benzonitrile, which stimulate the growth of *P. fluorescens* (Garbeva et al., 2014). BVCs can therefore provide positive information about surrounding microorganisms or environment. Alternatively, aerial exposure to glyoxylic acid and 2,3-butanedione, both produced by *B. subtilis* reduces *Burkholderia glumae*, *P. aeruginosa*, *Paenibacillus polymyxa* and *E. coli* surface motility (Kim et al., 2013). In the case of *E. coli*, this reduced motility correlates with the downregulation of 30 genes involved in chemotaxy and motility in *E. coli* (Kim et al., 2013). Several other BVCs such as 1-butanol, indole, 2-butanone or acetoin were also shown to influence *E. coli* and *P. aeruginosa* motility (Letoffe et al., 2014).

Bacterial volatile compounds cues also contribute to the development of bacterial community by influencing biofilm formation of Gram-negative and Gram-positive bacteria. Although still mechanistically unclear, volatile compounds such as indole, 1-butanol, 2-butanone, acetoin, ammonia, ethanol, hexadecane, glyoxylic acid, and trimethylamine display positive or negative influence on biofilm formation in one or several tested bacterial species (*B. subtilis, E. coli*, *P. aeruginosa*, and *Staphylococcus aureus)* (Letoffe et al., 2014). Recent studies also demonstrated that volatile acetic acid, a short-chain fatty acid, or ammonia can stimulate biofilm formation in *B. subtilis* and *S. aureus* (Nijland and Burgess, 2010; Letoffe et al., 2014; Chen et al., 2015). Whereas exposure to nitric oxide (NO) can positively affects biofilm formation of *Shewanella oneidensis*, *Azospirillum brasilense* or *Vibrio harveyi* (Henares et al., 2013; Barraud et al., 2014), it triggers biofilm dispersion in several Gram-negative and positive bacteria including *P. aeruginosa*, *E. coli*, *V. cholerae*, *B. licheniformis*, *S. marcescens*, *Fusobacterium nucleatum* (Barraud et al., 2009b), *S. woodyi* (Liu et al., 2012)*, S. enterica* (Marvasi et al., 2014), and *Neisseria gonorrhoeae* (Potter et al., 2009). In *P. aeruginosa*, the dispersing role of NO could be correlated to degradation of cyclic-di-GMP, a bacterial small molecule playing a central role in the switch between biofilm and planktonic lifestyle (Barraud et al., 2009a; Liu et al., 2012).

The development of high cell density bacterial communities can also lead to the accumulation of organic and inorganic BVCs altering bacterial environment and triggering response to different stresses, including exposure to antibiotics (Heal and Parsons, 2002). For instance, ammonia emitted by bacterial population increases at a distance resistance to tetracycline and ampicillin, and decreases resistance to aminoglycosides in all tested Gram-negative and Gram-positive bacteria exposed to ammonia (Bernier et al., 2011). In *E. coli*, ammonia mode of action involved its import through the AmtB channel followed by an increase in polyamine synthesis leading to modulation of antibiotic resistance profiles (Bernier et al., 2011). Interestingly, at a distance alkalinization of bacterial growth medium (up to pH 8.5) upon exposure to volatile ammonia was reported and involved in the increased resistance to ampicillin of *S. marcescens* and *S. rubidaea* (Cepl et al., 2014). Similarly, volatile trimethylamine (TMA), produced by reduction of trimethylamine-oxide (TMAO) in TMAO-rich environments such as animal gut and tissues (Barrett and Kwan, 1985; Bos et al., 2013), can also modulate bacterial resistance to several classes of antibiotics through medium alkalinization that affects proton motive force and membrane permeability (Letoffe et al., 2014).

Another inorganic BVC produced by many bacteria, hydrogen sulfide (H_2_S), confers multidrug resistance upon different pathogens (*B. anthracis*, *P. aeruginosa*, *S. aureus*, and *E. coli*) under aerobic conditions via the mitigation of oxidative stress induced by antibiotic treatment upon suppression of DNA-damaging Fenton reaction (Gusarov et al., 2009). Exposure to volatile 2,3-butanedione and glyoxylic acid, both naturally produced by *B. subtilis* GB03, alter *E. coli* antibiotic resistance profiles, which could be correlated to the upregulation of *hipA*, encoding an anti-toxin module previously described as mediating persistence (Kim et al., 2013). Alteration of antibiotic resistance by BVCs can also occur at the level of persistence. Indeed, volatile 2-amino-acetophenone (2-AA) enhances antibiotic tolerance by increasing accumulation of persistent bacteria in *P. aeruginosa* and *B. thailandensis* but also in the non-2-AA producer *Acinetobacter baumanii* (Que et al., 2013), two pathogens isolated during co-infection with *P. aeruginosa*. Since 2-AA promotes persistence by altering bacterial translation, an highly conserved machinery, and it can affect both producing and non-producing bacteria, this suggests that volatile 2-AA could be involved in the ability of Gram-negative bacteria to tolerate antibiotic treatment in polymicrobial infections.

Finally, *P. putida* exposure to indole produced by *E. coli* induces an eﬄux pump leading to an increased antibiotic resistance (Molina-Santiago et al., 2014). However, although it is well established that soluble indole influences drug resistance in several Gram-negative bacteria (Hirakawa et al., 2005; Lee et al., 2008, 2009; Nikaido et al., 2008; Molina-Santiago et al., 2014), its role as a significant airborne signal affecting drug resistance still needs to be confirmed.

## Concluding Remarks

### BVCs, an Untapped Pool of Bioactive Compounds?

Beyond its fundamental ecological interest, a better understanding of BVC roles, biosynthesis pathways and mechanisms of action could provide new information on the extent of bacterial metabolic potential and lead to clinical or industrial applications (**Figure 1**). Indeed, several soil-associated bacteria were not only shown to have positive effects on plant resistance but also to control plant diseases by exhibiting antibacterial activity against plant pathogens (Berg, 2009; Pieterse et al., 2014). BVCs can also influence bacterial pathogenesis by altering the production of virulence factors (i.e., 2,3-butanediol increasing virulence factor production in *P. aeruginosa*) or by affecting host cell functions (i.e., colonic homeostasis, T- and B cell proliferation responses or cytokine production; Kurita-Ochiai et al., 1995; Smith et al., 2013; Venkataraman et al., 2014).

Considering bacterial potential for metabolic adaptation to available environmental resources, characterization of the volatile secondary metabolites produced in nature could provide leads for the development of diagnostic tool using BVC as potential biomarker in some pathological situations (Probert et al., 2009). However, most bacteria releasing complex blends of molecules, unraveling the chemical nature and roles of BVCs emitted in mixed-species contexts will certainly constitute a major challenge of the field.

### Laboratory Conditions vs. Nature: A True Biological Functions for BVCs?

In the studied described above, experimental set-up using physically separated source of volatile compounds and recipient bacteria unambiguously demonstrated that exposure to BVCs could have important biological functions. While some highly abundant BVCs are likely to play a role in intra- and inter- bacterial competition and cooperation phenomena, most, if not all studies were performed in laboratory conditions, using artificial media and controlled temperature, atmosphere and BVC concentrations of unknown physiological relevance. Moreover, although BVC-dependent interactions between bacteria (and also plants, fungi, nematodes) are potentially occurring in environments such as soil or mammalian intestines, the high solubility of BVCs in the liquids present in these environments raises the question of the true aerial nature of BVC-mediated impact on bacteria. Future work will therefore have to clarify the role played by BVCs in bacterial ability to adapt and/or respond to their environments by determining the physiological concentrations of relevant BVCs in diverse environments and to establish, preferentially *in vivo*, the importance of airborne bacterial interactions in microbial ecology.

## Conflict of Interest Statement

The authors declare that the research was conducted in the absence of any commercial or financial relationships that could be construed as a potential conflict of interest.
